# METTL14 and WTAP play a crucial role in the regulation of bovine preadipocyte differentiation

**DOI:** 10.1080/10495398.2025.2476531

**Published:** 2025-03-17

**Authors:** Jia Liu, Chicheng Ma, Yu Cheng, Minzhi Wang, Guoqing Zhao, Liwei Huang, Ruigao Song, Xi Wang, Hongxia Li

**Affiliations:** ^a^ College of Animal Science, Shanxi Agricultural University, Taigu, Shanxi, China; ^b^ Reproductive Medicine Center, Department of Obstetrics and Gynecology, Shanxi Bethune Hospital, Shanxi Academy of Medical Sciences, Third Hospital of Shanxi Medical University, Tongji Shanxi Hospital, Taiyuan, China; ^c^ Shanxi Academy of Advanced Research and Innovation, Taiyuan, China

**Keywords:** Bovine, Preadipocytes, *METTL14*, *WTAP*, Cell differentiation

## Abstract

m6A methylation is the most common mRNA modification in mammals and plays a significant role in regulating various biological functions. Some studies have demonstrated that the methyltransferase *METTL3* can promote adipogenesis. However, the regulatory mechanisms of *METTL14* and *WTAP*, both methyltransferases, in adipogenesis remain unclear. This study investigated their effects on bovine preadipocyte differentiation using siRNA-mediated knockdown combined with transcriptomic analysis. Silencing *METTL14* and *WTAP* significantly impaired lipid droplet formation and revealed distinct regulatory pathways: *METTL14* knockdown affected genes like *JAK2* and *STAT3*, while *WTAP* suppression down-regulated *PPARγ/FABP4* signalling pathway components. These findings demonstrate that *WTAP* specifically modulates bovine adipocyte differentiation through the *PPARγ/FABP4* pathway.

## Introduction

The ability of beef cattle to deposit fat is closely related to factors such as breed, environment, nutritional level, gender and hormones. Yaks, adapted to plateau environment, consume fat to generate heat, resulting in significantly lower fat content in their meat compared to other beef varieties. The intramuscular fat content in yak meat ranges from 1.6% to 4.7%, typically increasing with age.^1^ Japanese Wagyu beef is characterized by its ability to accumulate a significant amount of intramuscular fat.^2^ The intramuscular fat content in Wagyu beef can also be enhanced by extending the fattening period.^3^ Additionally, dietary conditions play a crucial role in the deposition of intramuscular fat. Specifically, reducing dietary vitamin A intake during the fattening period of Wagyu cattle increases in intramuscular fat,^4^ while the addition of vitamin C has been shown to enhance marbling.^5^ Incorporating niacin^6^ and daidzein^7^ into the diet can boost intramuscular fat content in Angus cross cattle. Beyond dietary factors, fat deposition capability in beef cattle is also regulated by various mechanisms, including signalling pathways such as *PPARγ*, *WNT* and *AMPK*, as well as receptor proteins and transcription factors like *CD36*, *SREBP1* and *FABP4*.

The m6A methylation level of RNA is dynamically and reversibly regulated by a series of enzymes.^8^^,^^9^ The methyltransferase complex, including *METTL3/14* and *WTAP*, acts as the ‘Writer’, facilitating the addition of methyl groups to mRNA bases and catalysing the methylation reaction. Demethylases, such as *FTO* and *ALKBH5*, serve as ‘erasers’ by removing the m6A modification from mRNA. Binding proteins like *YTHDF1*, *YTHDF2* and *YTHDF3*, function as ‘readers’, recognizing and binding to m6A methylation sites to enable the modification to perform its biological functions effectively.

As an m6A demethylase, *FTO* is closely associated with lipid accumulation in various cells and tissues. Analysis of *FTO* expression in subcutaneous adipocytes from both obese and lean individuals revealed a positive correlation between *FTO* levels and body weight as well as body fat.^10^ In mouse and pig preadipocytes, *FTO* inhibits adipogenesis through the *JAK2-STAT3-C/EBPβ* signalling pathway. Reduced *FTO* expression leads to decreased *JAK2* and *STAT3* phosphorylation, resulting in weakened *C/EBPβ* transcription. *YTHDF2* directly targets *JAK2* mRNA, accelerating its decay and thereby reducing *JAK2* expression and inactivation the *JAK2-STAT3-C/EBPβ* signalling pathway.^11^ *WTAP* regulates gene expression at the post-transcriptional level by participating in the catalytic process of m6A modification, thereby influencing the deposition and characteristics of intramuscular fat.^12^

*METTL3* and *METTL14* also play roles in regulating lipid accumulation. *METTL3*-mediated m6A methylation destabilizes mRNA of metabolism-related genes, leading to metabolic disorders and lipid accumulation in the liver.^13^ Knockout of *METTL14* can improve fat deposition in the liver of mice fed a high-fat diet.^14^ Knocking down *METTL3* in cardiomyocytes further reduces triglyceride deposition.^15^ *FTO*, *METTL3* and *METTL14* can affect lipids by regulating fatty acid synthase (*FASN*), acetyl-CoA carboxylase *(ACCY*), stearoyl-CoA desaturase 1 (*SCD1*) and sterol regulatory element binding protein (*SREBP*) synthesis.^9^ *METTL16* plays a crucial role in the development of chicken muscle by promoting myoblast proliferation and inhibiting differentiation, while also regulating myofiber type formation and the overall m6A RNA methylation state.^16^

These studies underscore the close relationship between m6A methylation and fat synthesis. However, the mechanisms by which *METTL14* and *WTAP* influence bovine adipogenesis still require further investigation. Through the above, we can initially screen bovine species with high-fat deposition, subsequently introduce signal pathway activators or inhibitors to target m6A modification and downstream fat synthesis networks and ultimately integrate epigenetic regulation with nutritional metabolism programming. This approach allows for the optimization of fattening strategies in a staged manner. In the future, it will be essential to further analyse the cattle-specific regulatory network to facilitate the transition from molecular mechanisms to industrial applications.

## Materials and methods

### Source of bovine preadipocytes

The tissue samples and precursor adipocytes utilized in this study were sourced from the subcutaneous adipose tissue of three healthy Jinnan cattle (*n* = 3). Initially, the subcutaneous adipose tissue designated for primary cell isolation was rinsed with 75% alcohol on its surface. The tissue was then washed twice in DPBS (Gibco, New York, USA) containing dual antibodies (4% penicillin–streptomycin) and stored in DPBS with dual antibodies (1% penicillin–streptomycin). The centrifuge tube was sealed with a sealing film and transported at low temperature. The laboratory promptly commenced the isolation and extraction of preadipocytes. This study was executed under the approval of the Institutional Animal Care and Use Committee of Shanxi Agricultural University.

### Bovine preadipocyte extraction

Wash the tissue twice more with DPBS containing 1% penicillin–streptomycin on a clean workbench. Next, cut the tissue into pieces and collect them into a centrifuge tube. Add an appropriate amount of collagenase (1 mg/mL) for digestion. Terminate the digestion by adding an equal volume of culture medium. The digestive solution was filtered through a 70 μm nylon mesh, and the filtrate was collected into centrifuge tubes, followed by centrifugation at 1500 rpm for 5 min. The supernatant was discarded, and complete medium [composed of 10% FBS (Sangon Bioengineering), 1× penicillin–streptomycin dual antibiotics and DMEM/F12 (Gibco) basal medium] was added. After thorough mixing by pipetting, the solution was plated onto 10 cm culture dishes and placed in a 37 °C, 5% CO_2_ incubator for cultivation. The medium was replaced every 2 days.

### Induced differentiation of preadipocytes

Bovine preadipocytes were cultured in a six-well plate. Once the cell confluence in the well plate reached approximately 70%, the complete medium was replaced with differentiation medium [take the preparation of 50 mL as an example, 5 mL FBS, 0.5 mL 100× PS, 1 mL Hepes (IM), 50 μL Insulin (5 mg/mL), 50 μL Biotin (33 mM), 25 μL IBMX (0.5 M), 50 μL pantothenic acid (17 mM), 2 μL Rosi (25 mM), 0.5 μL DEX (10 mM), add high-glucose DMEM medium to 50 mL], with medium changes occurring every day. After 2 days of differentiation, the differentiation medium was substituted with mature medium [take the preparation of 50 mL as an example, 5mL FBS, 0.5 mL 100× PS, 1mL Hepes (IM), 50 μL Insulin (5 mg/mL), 50 μL Biotin (33 mM), 50 μL pantothenic acid (17 mM), 0.5 μL DEX (10 mM), add high-glucose DMEM medium to 50 mL)] and the medium was changed every 2–3 days based on the condition of the cells.

### siRNA sequence synthesis and cell transfection

In this experiment, the siRNA was synthesized by Sangon Bioengineering (Shanghai, China). Two siRNAs were synthesized for both *METTL14* and *WTAP*, along with one negative control (Table 1).

**Table 1.** siRNA sequence information.

Name	Sense (5′–3′)	Antisense (5′–3′)
*si-METTL14-541*	CGGACAGAUUUGAAGAAUATT	UAUUCUUCAAAUCUGUCCGTT
*si-METTL14-708*	GAAGAAUAUUACAGAGAGATT	UCUCUCUGUAAUAUUCUUCTT
*si-WTAP-310*	GCAAGUACACAGAUCUCAATT	UUGAGAUCUGUGUACUUGCTT
*si-WTAP-248*	GGCACGAGAUGAAUUAAUUTT	AAUUAAUUCAUCUCGUGCCTT
NC	UUCUCCGAACGUGUCACGUTT	ACGUGACACGUUCGGAGAATT

In this experiment, the lipofectamine transfection method was employed to introduce siRNA, following the instructions provided by the Lipofectamine 3000 transfection kit (Invitrogen, California, USA). Each group of siRNA was prepared in triplicate. The transfection of siRNA was conducted in a six-well plate. After the cells were passaged, typically 12–24 h later, they were deemed ready for transfection when the confluence of bovine preadipocytes reached 60–80%. RNA or protein samples from the cells can be collected as needed 48 h post-transfection.

### RNA extraction

In this experiment, total RNA was extracted from adipocyte samples using the Trizol method. Trizol reagent was added directly to the six-well plate to lyse the cells. We opted not to use trypsin for cell digestion before centrifugation and transfer to a 1.5 mL centrifuge tube because trypsin can adversely affect RNA extraction and quality, potentially causing RNA degradation during digestion and centrifugation. To mitigate degradation risks, it is crucial to perform the RNA extraction promptly, particularly after RNA precipitation, to avoid RNA degradation at room temperature.

### Total RNA reverse transcription

RNA reverse transcription was performed according to the instructions provided with Severn’s innovative reverse transcription kit (All-in-one First Strand cDNA Synthesis Kit II, with dsDNase). Based on the concentration of the sample RNA, an appropriate amount of total RNA (approximately 1 μg) was prepared, and the reaction system was set up on ice. The mixture was gently mixed by pipetting, centrifuged briefly and then transferred to a PCR machine for incubation at 37 °C for 2 min to remove genomic DNA contamination. Following this, the mixture was incubated at 42 °C for 10–20 min, then at 85 °C for 5 s to terminate the reaction. The resulting product can be directly utilized for second strand synthesis or subsequent qPCR amplification. Typically, the cDNA product has a high concentration and can be diluted four to five times with Nuclease-Free water for future applications, with storage recommended at −20 °C.

### Real time fluorescence quantitative PCR

Based on the gene transcript information on NCBI, qPCR primers were designed. The primers sequence information are shown in Table 2.

**Table 2.** qPCR Primer sequence information.

Gene	Product length	Sequence (5′–3′)
β-ACTIN	148	F primer: GCGGCATTCACGAAACTACC
		R primer: GCCAGGGCAGTGATCTCTTT
*METTL14*	194	F primer: TAAAGCGTAGCACAGACGGG
		R primer: AACTGTAAGCCACCCTGGTC
*WTAP*	140	F primer: GCTTTGGAGGGCAAGTACAC
		R primer: TGCTCCTTGGTTGCTAGTCG

After obtaining cDNA through reverse transcription, prepare a qPCR reaction system and set up three replicates for each primers pair. Calculate the gene expression levels using the (2^−ΔΔCT^) method and analyse the differences based on the CT values obtained.

### RNA sequencing

After 48 h of siRNA transfection in bovine preadipocytes, total RNA was extracted from two transfected groups and one control group, with each group consisting of three replicate. Samples were stored at −80 °C and transported on dry ice. Transcriptome sequencing was performed by Shanghai Sangon Bioengineering Company.

### Statistical analyses

Data analysis and graph generation were conducted using GraphPad Prism 8.0. *P*-Values were calculated using an unpaired *t*-test. Results are presented as the mean ± standard error of the mean (SEM) from experiments conducted in triplicate(*P < 0.05; **P < 0.01).

## Results

### Determination of optimal transfection efficiency of METTL14 and WTAP

Bovine preadipocytes were cultured in 24-well plates and transfected when the cell confluence reached approximately 70%. The study comprised five groups in total, categorized into four siRNA groups, *si-METTL14-708* (hereinafter referred to as *si-M708*), *si-METTL14-541*, *si-WTAP-248* and *si-WTAP-310* (hereinafter referred to as s*i-W310*), along with a negative control (NC) group, with three replicates established for each group.

**Figure 1.**
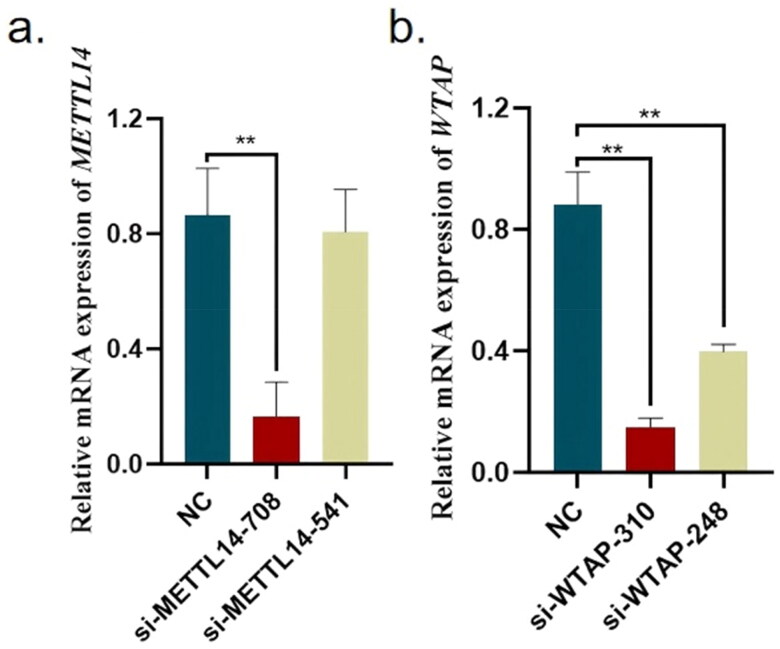
qPCR result of *METTL14* and *WTAP*. Data are presented as mean ± SEM; *n* = 3 per group. *P < 0.05; **P < 0.01.

The analysis revealed that the *si-M708* group exhibited the highest interference efficiency, with the *METTL14* expression level reduced to only 19.194% of that observed in the NC group. For *WTAP* siRNA, both tested groups demonstrated high interference efficiency. Specifically, the *si-W310* group showed a *WTAP* expression level reduced to 16.854% of the NC group, while the *si-WTAP-248* group exhibited a *WTAP* expression level of 50.594% relative to the NC group, as illustrated in Fig. 1. Based on these findings, *si-M708* and *si-W310* were selected for subsequent interference experiments.

### *Interference with* METTL14 *and* WTAP *significantly inhibits lipid accumulation in bovine preadipocytes*

Bovine preadipocytes were transfected with *si-M708* and s*i-W310*, respectively. Forty-eight hours after transfection, both the transfected and NC groups were induced to differentiate. After an additional 48 h of differentiation, the cells were switched to maturation medium, with this day designated as day 0. The differentiation of bovine adipocytes was then observed and recorded daily.

**Figure 2.**
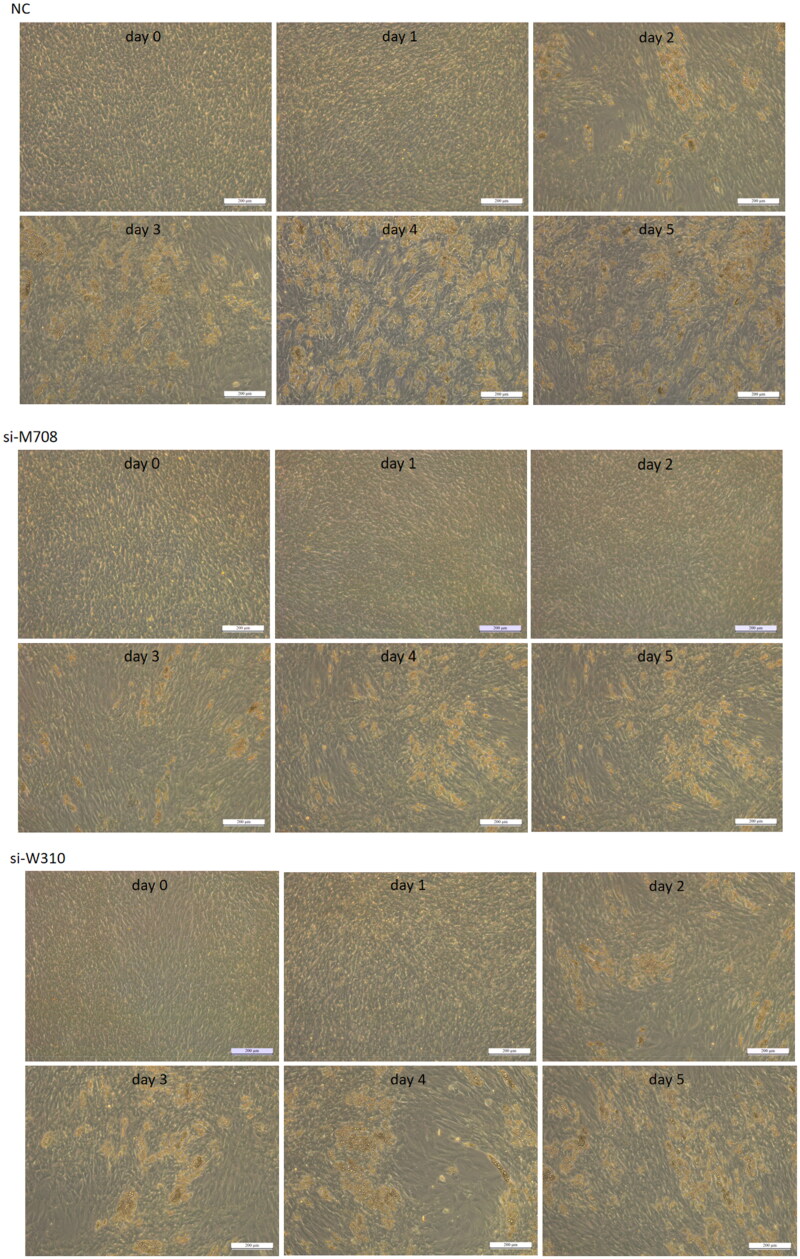
Differentiation of preadipocyte in cattle (scale bar = 200 μm).

Figure 2 illustrates that bovine preadipocytes across the three groups exhibited a rounded cell morphology on day 0. By day 1, cells in the NC group and s*i-W310* group began to show individual small lipid droplets, while the *si-M708* group did not show small lipid droplets until day 2. On day 2, extensive areas of lipid droplets were observed in the NC and s*i-W310* groups, while the *si-M708* group only exhibited small lipid droplets differentiation on day 3. By day 5, preadipocytes in all three groups were capable of aggregating small intracellular lipid droplets into larger droplets, ultimately forming large-area lipid droplets.

**Figure 3.**
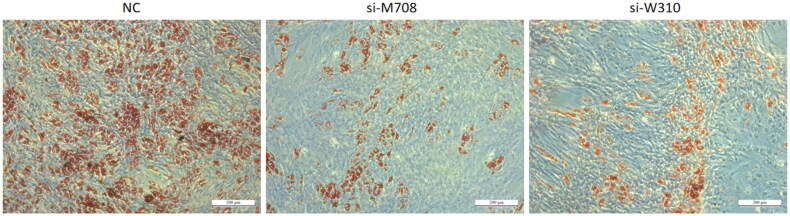
ORO staining of preadipose cells in cattle after differentiation (scale bar = 200 μm).

On day 6, the large-area lipid droplets that had formed were stained with Oil Red O. As shown in Fig. 3, the number of lipid droplets in the *si-M708* and s*i-W310* groups was significantly lower compared to the NC group, indicating that the interference with the expression of *METTL14* and *WTAP* can significantly reduce lipid accumulation and the differentiation capabilities of bovine preadipocytes.

### *Differential gene analysis of bovine preadipocytes transfected with* si-METTL14

**Figure 4.**
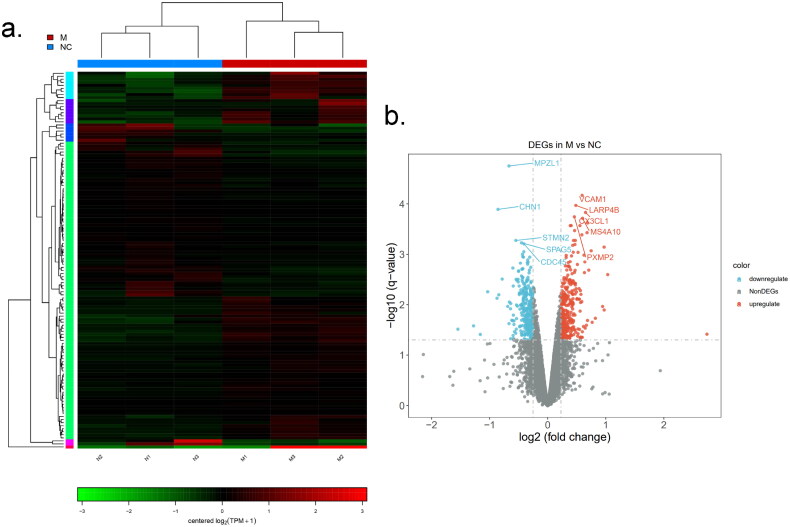
Screening of *si-METTL14* DEGs: (a) heat map of sample correlation between NC and M groups. Red represents a positive correlation, while green represents a negative correlation; (b) the volcanic map of DEGs. The abscissa is the fold change of genes between NC and M groups, the ordinate is corrected *P*-value of the difference in genes level.

Interference with *METTL14* expression can significantly impair the differentiation capability of preadipocytes and decrease lipid accumulation. However, the specific regulatory mechanisms and impacts of *METTL14* on adipocyte differentiation remain unclear. To elucidate this, we employed RNA sequencing (RNA-seq) technology to investigate the regulatory role of *METTL14* in bovine preadipocytes. As illustrated in Fig. 4a, we identified 124 differentially expressed genes (DEGs), with 65 genes being up-regulated and 59 down-regulated. Notably, genes such as *VCAM1*, *CX3CL1*, *CHN1*, *MPZL1* and *STMN2* exhibited highly significant differences (*P* < 0.01), as presented in Fig. 4b.

### *Functional analysis of DEGs GO and KEGG enrichment analysis in bovine preadipocytes transfected with* si-METTL14

**Figure 5.**
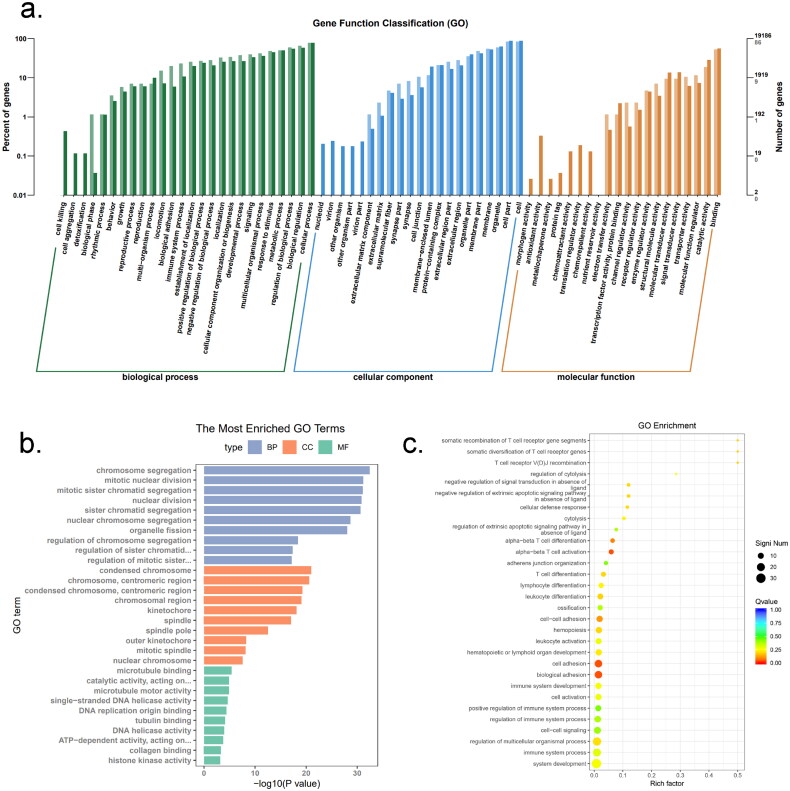
GO functional analysis of *si-METTL14* DEGs (a) GO function classification each column represents a multilevel classification of GO. The abscissa represents the multilevel classification term of GO. The left and right ordinates represent the percentage of the gene contained in the classification to total number of genes and the number of genes in the classification, respectively; (b) the most enrichment GO terms. The abscissa is the corrected *P*-value, the ordinate is the descriptive information of GO functions; (c) GO enrichment bubble chart. The abscissa is the function annotation information, the ordinate is the rich factor corresponding to the function.

To further investigate the functions of *METTL14* differential genes, we conducted Gene Ontology (GO) functional analysis and Kyoto Encyclopedia of Genes and Genomes (KEGG) enrichment analysis on the identified DEGs. The analysis of the most enriched GO functions revealed that a substantial number of *METTL14* DEGs are involved in regulating biological processes such as chromosome segregation, mitosis and sister chromatid separation. Additionally, most DEGs are associated with cellular components, particularly chromosome regions and centromere regions. A smaller subset of DEGs is linked to molecular functions, including single-stranded DNA helicase activity, DNA replication origin binding and ATP-dependent activities, as illustrated in Fig. 5a,b. Furthermore, the bubble chart of GO functional enrichment presented in Fig. 5c indicates that DEGs are significantly enriched in functions related to T cell differentiation, cell adhesion and bioadhesion.

**Figure 6.**
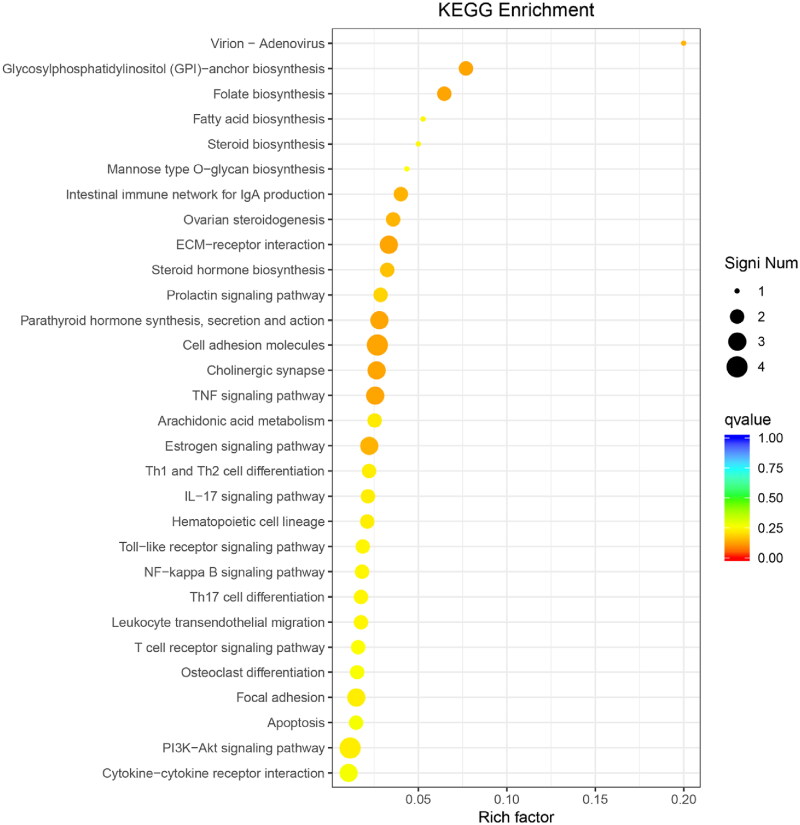
KEGG functional analysis of *si-METTL14* DEGs. The ordinate is the pathway name, the abscissa is the corresponding rich factor for each pathway.

Additionally, as shown in Fig. 6, DEGs were significantly enriched in pathways related to cell adhesion, oestrogen signalling, *TNF* signalling, *JAK-STAT3* signalling and the *PI3K-AKT* signalling pathway. A smaller subset of DEGs was also found to be enriched in the cholesterol synthesis and fatty acid synthesis pathways. The results indicated that the knockout *METTL14* could affect the synthesis of fatty acids, which was consistent with the phenotypic results mentioned above. In summary, it appears that a limited number of DEGs related to *METTL14* may directly influence and regulate the pathways involved in fat synthesis and differentiation. Coupled with the phenotypic observations of reduced lipid accumulation, it is hypothesized that *METTL14* likely impacts the upstream pathways governing lipid synthesis and fat differentiation, thereby indirectly regulating fat deposition in preadipocytes.

### The qPCR results of si-METTL14 transfected bovine preadipocytes DEGs

**Figure 7.**
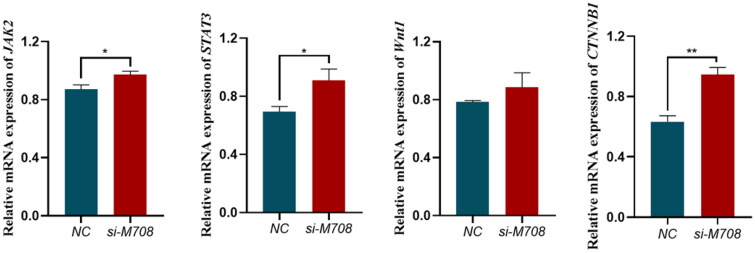
qPCR Result of si-METTL14 DEGs. Data are presented as mean ± SEM; *n* = 3 per group. *P < 0.05; **P < 0.01.

Combining GO functional enrichment analysis and KEGG pathway enrichment analysis, screen the DEGs obtained from RNA-seq. Select key regulatory factors on the *JAK-STAT3* pathway: *JAK*, *STAT3*, *WNt1* and *CTNNB1*. To explore the regulatory mechanism of *METTL14* on fat deposition by measuring its expression level through qPCR. The results of qPCR are shown in Fig. 7. The decrease in *METTL14* expression level significantly affects the expression of *JAK2, STAT3* and *CTNNB1*. The expression of Wnt1 gene showed a similar trend, although the results were not significant.

### Differential gene analysis of bovine preadipocytes transfected with si-WTAP

**Figure 8.**
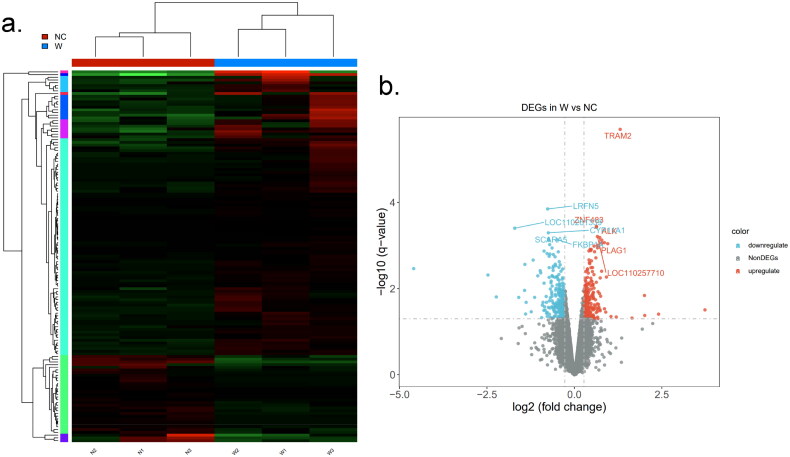
Screening of *si-WTAP* DEGs: (a) heat map of sample correlation between NC and W groups. Red represents a positive correlation, while green represents a negative correlation; (b) the volcanic map of DEGs. The abscissa is the fold change of protein expression between NC and W groups, the ordinate is corrected *P*-value of the difference in protein expression level.

According to the results of previous cell experiments, the lipid droplets formed by preadipocytes in the induced differentiation s*i-W310* group were found to be significantly fewer than those in the NC group. This indicates that interference with *WTAP* has a substantial impact on lipid synthesis and adipocyte differentiation; however, the specific regulatory mechanism of *WTAP* on adipocyte differentiation remains unclear. Therefore, we employed RNA-seq technology to simultaneously investigate the potential regulatory mechanism of *WTAP* on lipid synthesis. Fig. 8a illustrates the expression levels of DEGs associated with *WTAP*. A total of 137 DEGs were identified through RNA-seq, with 104 DEGs being up-regulated and 33 DEGs down-regulated. Notably, genes such as *TRAM2*, *ALK*, *PLAG1*, *LRFN5*, *SCARA5* and *FKBP1* exhibited extremely significant differences (*P* < 0.01), as depicted in Fig. 8b.

### *Functional analysis of DEGs GO and KEGG enrichment analysis in bovine preadipocytes transfected with* si-WTAP

**Figure 9.**
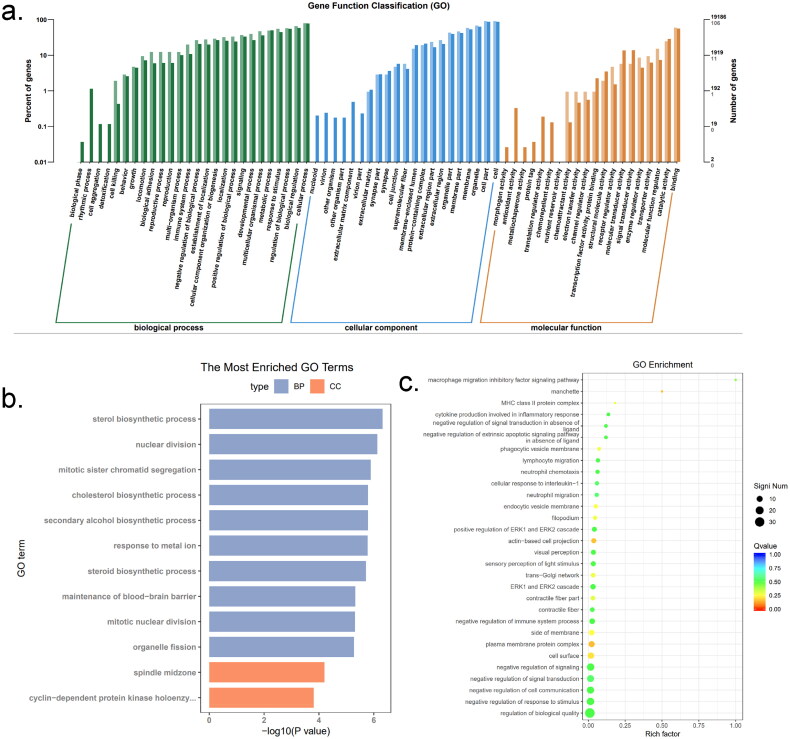
GO functional analysis of *si-WTAP* DEGs: (a) GO function classification each column represents a multilevel classification of GO. The abscissa represents the multilevel classification term of GO. The left and right ordinate represent the percentage of the gene contained in the classification to total number of genes and the number of genes in the classification, respectively; (b) the most enrichment GO terms. The abscissa is the corrected *P*-value, the ordinate is the descriptive information of GO functions; (c) GO enrichment bubble chart. The abscissa is the function annotation information, the ordinate is the rich factor corresponding to the function.

To further investigate the functions of the DEGs associated with *WTAP*, GO functional analysis and KEGG enrichment analysis were conducted on the identified DEGs. As illustrated in Fig. 9a,b, a greater number of DEGs are found in cellular components and biological processes. The analysis of the most enriched GO functions revealed that a significant proportion of *WTAP* DEGs are involved in biological processes such as sterol biosynthesis, nuclear division, separation of mitotic sister chromatids, response to metal ions and maintenance of the blood-brain barrier. Conversely, a smaller number of DEGs are associated with cellular components, including protein kinase-dependent and spindle-centred components, while very few DEGs pertain to molecular functions. The bubble plot of GO functional enrichment presented in Fig. 9c highlights that DEGs are significantly enriched in functions such as plasma membrane protein complexes, cytoskeleton organization, positive regulation of *ERK1* and *ERK2* signalling cascades and negative regulation of signalling pathways.

**Figure 10.**
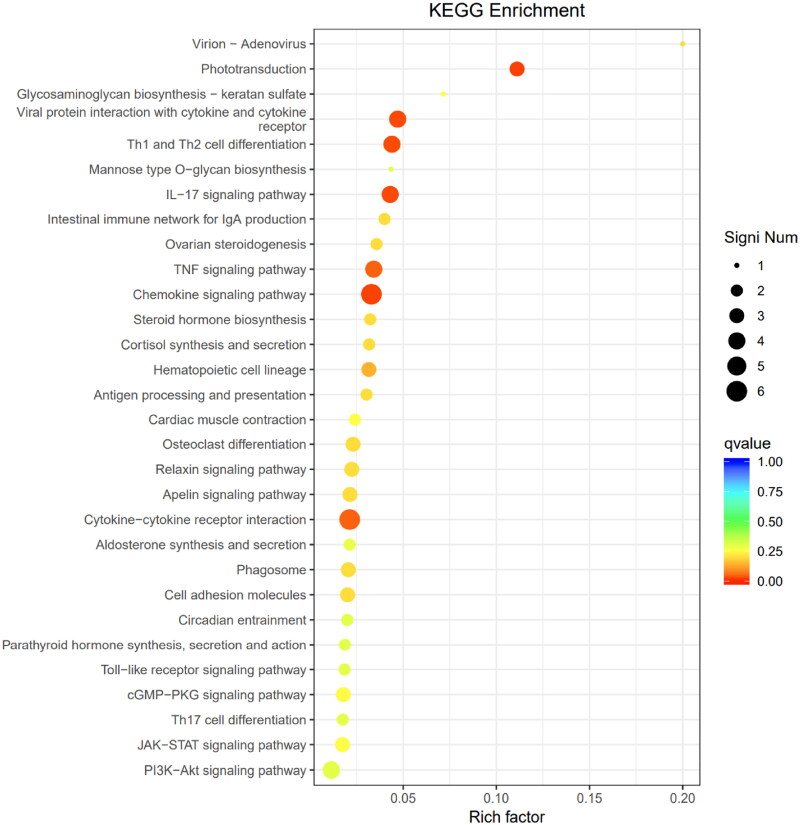
KEGG functional analysis of *si-WTAP* DEGs. The ordinate is the pathway name, the abscissa is the corresponding rich factor for each pathway.

The enrichment of DEGs in the secondary pathways is presented in Fig. 10. Notably, DEGs are significantly enriched in pathways related to cytokines and cytokine receptor interactions, phototransduction, ovarian steroid synthesis, Th1 and Th2 cell differentiation, chemokine signalling and the *PI3K-AKT* signalling pathway. In summary, it can be concluded that the DEGs related to *WTAP* do not directly act upon or regulate the pathways of adipogenesis. Coupled with the phenotypic results demonstrating reduced lipid accumulation following the induced differentiation of early adipocytes, it is speculated that the regulatory mechanism of *WTAP* resembles that of m6A methyltransferases. Specifically, it is likely akin to *METTL14*, functioning within the upstream pathways of lipid synthesis and adipogenesis, such as the *PI3K-AKT* signalling pathway, to indirectly regulate fat deposition in preadipocytes.

### The qPCR results of si-WTAP transfected bovine preadipocytes DEGs

**Figure 11.**
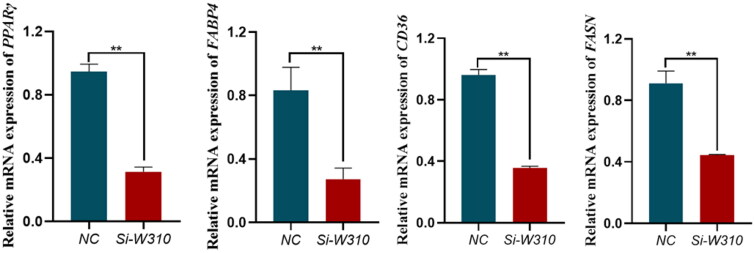
qPCR result of si-METTL14 DEGs. Data are presented as mean ± SEM; *n* = 3 per group. *P < 0.05; **P < 0.01.

Combining GO functional enrichment analysis and KEGG pathway enrichment analysis, screen the DEGs obtained from RNA-seq. Select key regulatory factors on the *PPARγ* pathway and *AMPK* pathway: *PPARγ*, *FABP4*, *CD36* and *FASN*. To explore the regulatory mechanism of *WTAP* on fat deposition by measuring its expression level through qPCR. The qPCR results are shown in Fig. 11. After knocking down the expression level of *WTAP*, the expression of the marker regulatory factor *PPAR γ* on the *PPAR γ* pathway decreased significantly, and the expression of *FABP4* was also significantly reduced. The expression of *CD36* and *FASN* was also significantly reduced, which is consistent with the sequencing results.

## Discussion

Beef cattle breeding plays a crucial role in livestock production. The proportion of beef in the meat market is steadily increasing, and the quality of beef has gradually become a focal point of market interest. High-quality beef is typically characterized by marbling, with the intramuscular fat content directly influencing the development of marbling.^17^ Yamada et al. proposed a model for intramuscular fat deposition, suggesting that energy is first accumulated in visceral fat tissue. Once the energy reaches the upper limit of visceral fat storage, it is redirected to subcutaneous and intramuscular fat, thereby promoting muscle growth and internal fat deposition. The regulatory mechanisms affecting fat deposition are complex, as the interplay between signalling pathways and transcription factors results in a non-linear regulatory framework. Common signalling pathways involved in lipogenesis and adipogenesis include *PPARγ*, *WNT* and JAK/STAT, with marker genes such as *c-MYC*, *CTNNB1*, *FABP4*, *SREBP1* and *FASN*.m6A methylation modification is the most prevalent mRNA modification in mammals and plays a significant role in various biological processes.^18^ Numerous studies have demonstrated that m6A modification is involved in the regulation of fat synthesis, lipid metabolism and other physiological activities.^19^ The *FTO* gene, first identified in 2011, is associated with obesity and is highly expressed in adipose tissue and the hypothalamus.^8^ Research has shown that *FTO* functions as a demethylase in m6A modification, promoting fat synthesis and storage, facilitating lipid accumulation in the liver and inhibiting lipid metabolism.^20^ *METTL3* is a methyltransferase involved in m6A modification. Studies have demonstrated that *METTL3*-mediated m6A methylation destabilizes the mRNA of metabolism-related genes, contributing to metabolic disorders and lipid accumulation in the liver.^13^ Similarly, *METTL14* and *WTAP*, which were identified concurrently with *METTL3* as m6A methyltransferases, also influence lipogenesis, lipid metabolism and deposition in animals.^9^ Adipocyte differentiation is regulated by a variety of mechanisms, among which mitotic clonal expansion is a critical step, and *WTAP* and *METTL14* induce increased and distributed in the nucleus by regulating adipogenesis. In mice, knockdown of these two proteins has been found to lead to cell cycle arrest and impaired lipogenesis, as evidenced by decreased and decreased adipocytes.^21^ Additionally, *WTAP* has been shown to regulate lipid metabolism by recruiting both *METTL3* and *METTL14*.^9^

In this study, we determined that the capacity of bovine preadipocytes to accumulate lipids and differentiate into adipocytes was significantly diminished as the expression levels of *METTL14* and *WTAP* decreased. During the differentiation induction experiment, both the NC group and the s*i-W310* group exhibited large lipid droplets by day 2; however, the lipid droplets formed in the s*i-W310* group were significantly fewer than those in the NC group by the conclusion of the experiment. In the *si-METTL14* group, large lipid droplets emerged by day 3, but the final quantity of lipid droplets formed was substantially lower than that in the NC group. These findings indicate that reduced expression levels of *METTL14* and *WTAP* adversely affect the differentiation of preadipocytes.

The DEGs of *METTL14* are significantly enriched in the *JAK/STAT* and *Wnt* signalling pathways, with certain DEGs in these pathways identified as regulators of adipocyte progression.^22^ The *JAK/STAT* signalling pathway is widely expressed and is activated by cytokines, playing a crucial role in various physiological processes, including cell proliferation, differentiation and apoptosis.^23^ The downstream *STAT* family has been shown to regulate multiple functions in adipocytes, with *STAT1*, *STAT3*, *STAT5A* and *STAT5B* recognized for their essential roles in adipocyte generation and differentiation.^24^^,^^25^ Specifically, the *JAK2/STAT3* pathway influences preadipocyte differentiation by modulating the expression of *C/EBPβ*.^26^ The *WNT* pathway, a well-established signalling cascade, is known to regulate adipogenesis and is viewed as a pivotal switch in this process.^27^ The *WNT/β-Catenin* pathway is a key regulatory mechanism that has been demonstrated to influence myocardial glucose metabolism, lipid metabolism and mitochondrial oxidation.^28^

The DEGs of *METTL14* identified through RNA-seq results include *GHR*, *SOCS1* and *SFRP1. GHR* is a transcription factor that acts upstream of the *JAK/STAT3* pathway and has the ability to phosphorylate *STAT3*.^29^ *SOCS1* is a downstream target gene regulated by the *JAK* family. SFRP1 inhibits the regulation of downstream genes by the *WNT* protein family within the *WNT* signalling pathway.^30^ Key transcription factors in the *WNT* signalling pathway include *WNT1, β-Catenin* (*CTNNB1*)*, TCF7* and *Sirt1. β-Catenin*, a target gene of the *WNT* protein family, serves as a signature transcription factor for *WNT* in regulating fat synthesis and differentiation.^31^ It often influences downstream pathways and transcription factors such as *PPARγ* and *SREBP1* through its interaction with the *WNT/β-Catenin* axis.^32^

The screening of DEGs of *WTAP* has revealed that the DESs associated with adipocytes include GATA2 and CREB1, which are involved in the *PPARγ* and *AMPK* signalling pathways, respectively.^33^ *PPARγ* is the most well-known pathway regulating adipogenesis and differentiation. The *PPAR* pathway comprises three subtypes: *PPARα, PPARβ/δ* and *PPARγ*.^34^ Among these, *PPARα* and *PPARβ/δ* primarily regulate biological processes related to lipid metabolism, including lipid transport, lipogenesis, fatty acid oxidation and degradation.^35^^,^^36^ These pathways predominantly influence fat metabolism by modulating the expression of the upstream gene *FABP4* during adipocyte differentiation.^37^^,^^38^ Furthermore, *PPARγ* regulates the expression levels of *SCD*1,^39^ which is crucial for adipogenesis, as well as *FABP*s and *LPL*, which play significant roles in lipid transport.^40^ *AMPK*, or AMP-activated protein kinase, serves as a key sensor of cellular energy levels and is a major regulator of nutrient metabolism.^41^ Its signalling pathway is particularly important for fat deposition and glucose homeostasis in both animals and humans.^42^ The *AMPK/SIRT1* axis represents a crucial pathway through which *AMPK* regulates fat deposition. Some studies have indicated that, under the influence of vitamin D, the *AMPK/SIRT1* axis can enhance intramuscular fat deposition while simultaneously preventing mitochondrial dysfunction.^43^ Analysis of the KEGG signalling pathway diagram reveals that *AMPK* can indirectly inhibit the expression of *SREBP1*, suppress mTOR phosphorylation, promote *PPARγ* phosphorylation and indirectly activate *CD36* activity. *CD36* is notable for its high capacity for fatty acid metabolism and its role in facilitating fatty acid transport across various tissues.^44^^,^^45^ Furthermore, AMPK can also indirectly enhance the expression of *CD36*.

The differential genes *GATA2* and CREB1 identified through the analysis of RNA-seq results for *WTAP* are positioned upstream of the *PPARγ* pathway and downstream of the AMPK pathway, respectively. Some studies have indicated that the expression of *PPARγ* can be inhibited by enhancing the expression of *GATA2*.^46^ *C/EBPα* serves as the primary regulator of adipose tissue development^47^ and exhibits high expression levels in both liver and adipocytes.^48^ It indicates that m6A methyltransferase is involved in the regulation of adipocyte differentiation; however, the specific mechanisms of action remain complex and warrant further investigation.

The study establishes critical mechanistic insights into fat deposition—a trait directly tied to beef quality and market value. Firstly, by identifying *METTL14* and *WTAP* as key regulators of adipogenesis, this work provides actionable genetic targets for breeding programs. Genes including *FABP4* and *PPARγ*, which demonstrate prominently elevated expression levels in the relevant lipid metabolic pathways, hold the potential to serve as crucial biomarkers. These biomarkers can be instrumental in precisely identifying cattle that possess remarkable marbling potential, thus providing valuable insights for selective breeding strategies within the cattle.

## Conclusions

By interfering with the expression of the m6A methyltransferases *METTL14* and *WTAP*, we observed a reduction in the differentiation ability of bovine preadipocytes corresponding to decreased expression levels of these proteins. Analysis of RNA-seq results indicated that *WTAP* regulates the differentiation of bovine preadipocytes through the *PPARγ/FABP4* pathway. This study establishes a clear link between the m6A methyltransferases *METTL14* and *WTAP* and the differentiation capacity of bovine preadipocytes, elucidating the pathway by which *WTAP* influences adipogenesis in these cells.
